# When Chatbots Meet Patients: One-Year Prospective Study of Conversations Between Patients With Breast Cancer and a Chatbot

**DOI:** 10.2196/12856

**Published:** 2019-05-02

**Authors:** Benjamin Chaix, Jean-Emmanuel Bibault, Arthur Pienkowski, Guillaume Delamon, Arthur Guillemassé, Pierre Nectoux, Benoît Brouard

**Affiliations:** 1 Department of Otolaryngology Head and Neck Surgery, University Hospital Gui de Chauliac Montpellier France; 2 Université Montpellier I Montpellier France; 3 Wefight, Brain & Spine Institute, Hospital Pitié-Salpêtrière Paris France; 4 Department of Radiation Oncology, European Hospital Georges Pompidou, Assistance Publique-Hôpitaux de Paris Paris France

**Keywords:** artificial intelligence, breast cancer, mobile phone, patient-reported outcomes, symptom management, chatbot, conversational agent

## Abstract

**Background:**

A chatbot is a software that interacts with users by simulating a human conversation through text or voice via smartphones or computers. It could be a solution to follow up with patients during their disease while saving time for health care providers.

**Objective:**

The aim of this study was to evaluate one year of conversations between patients with breast cancer and a chatbot.

**Methods:**

Wefight Inc designed a chatbot (Vik) to empower patients with breast cancer and their relatives. Vik responds to the fears and concerns of patients with breast cancer using personalized insights through text messages. We conducted a prospective study by analyzing the users’ and patients’ data, their usage duration, their interest in the various educational contents proposed, and their level of interactivity. Patients were women with breast cancer or under remission.

**Results:**

A total of 4737 patients were included. Results showed that an average of 132,970 messages exchanged per month was observed between patients and the chatbot, Vik. Thus, we calculated the average medication adherence rate over 4 weeks by using a prescription reminder function, and we showed that the more the patients used the chatbot, the more adherent they were. Patients regularly left positive comments and recommended Vik to their friends. The overall satisfaction was 93.95% (900/958). When asked what Vik meant to them and what Vik brought them, 88.00% (943/958) said that Vik provided them with support and helped them track their treatment effectively.

**Conclusions:**

We demonstrated that it is possible to obtain support through a chatbot since Vik improved the medication adherence rate of patients with breast cancer.

## Introduction

### Background

According to the World Health Organization, improved adherence would have more impact in terms of global health than the development of new drugs [1]. In the field of cancer, noncompliance is a consequence of not only the toxicity of anticancer drugs but also the nature of new treatments: oral chemotherapies (50% of chemotherapies in 2020) [2,3] shift the responsibility for taking treatment from caregivers to patients. Finally, the number of cancer patients is increasing exponentially (32.6 million and +17 million per year) [4], and this disease is becoming chronic (50% of patients are alive after 5 years) [5]. Most cancer patients are treated at home and have to manage their treatment alone.

On the other side, information technology is on the rise and is changing the way patients and physicians interact together [6,7]. Technology-based self-service channels [8] and digital health interventions [9] have the potential to support patients all day long and connect them to medical staff thanks to smartphone apps or wearable devices [10].

A chat is a software that interact with users by using a decision map, an algorithm, without human back-end intervention. Chatbots could be a solution to follow up with patients during their treatments and save time for health care providers. They create a dynamic interaction, are easy to use, and simulate a human conversation through text or voice via smartphones or computers.

Chatbots’ conversational abilities quickly improve [11] and public interest grows [12]. Now, patients can interact to describe their symptoms, after which advice and information are given in return by chatbots. As an example, patients can use chatbots to check symptoms and monitor their mental health [13,14]. Ly et al [15] assessed the effectiveness and adherence of a smartphone app that delivers strategies used in positive psychology to improve happiness and reduce negative symptoms.

### Objectives

In this study, we have aimed to determine what the interactions are when a human chats with a chat robot. Lucas et al [16] show that people may feel more comfortable disclosing personal information to a chatbot compared with a person as chatbots do not think or form judgments of their own. We suggest that health chatbots should be evaluated so that they can be an integral part of the doctor/patient relationship. Indeed, very few articles deal with chatbots in general and even fewer deal with health care chatbots and their interaction with humans. As such, we think that chatbots are an effective way to tackle the problems patients with breast cancer are facing. To accomplish this, Wefight Inc designed a chatbot named Vik to empower patients with breast cancer and their relatives via personalized text messages. Vik’s answers are very diverse, and patients can find all the relevant, quality-checked medical information they need. Vik informs about breast cancer and its epidemiology, treatments and their side effects, and the quality of life, with information about sport, fertility, sexuality, and diet. More practical information, such as reimbursement and patients’ rights, is also available. The goal is to improve the quality of life of the patients with breast cancer.

## Methods

### Study Design

In this study, we analyzed the conversations between patients with breast cancer and the chatbot, Vik, and the way they are using it. We also observed whether a chatbot like Vik could reinforce medication adherence by using a prescription reminder through the conversation.

Vik is available for free on the Web or from any smartphone, iOS or Android, on Messenger [17]. Vik’s platform is designed to address current and future patients’ needs. Its architecture is composed of several technological parts, allowing a fine analysis of the questions posed by the patients and an adapted treatment of the answer. To understand the users’ messages and send personalized answers, the conversation goes through 3 steps: the first step analyzes the sentence and identifies intents and entities, using machine learning. The second stage activates modules according to the intents and entities detected by the first stage, and the third stage aggregates the answers of all activated modules to build the answer sent to the user and saves the conversation on the user’s profile.

The data collected are anonymized and then hosted by Wefight Inc. In accordance with the French and European laws on information technology and civil liberties (Commission Nationale Informatique et Libertés and Règlement Général pour la Protection des Données), users have a right of use at their disposal to verify its accuracy and, if necessary, to correct, complete, and update it. They also have a right to object to their use and a right to delete these data. General conditions of use are displayed and explained very clearly; they must be accepted before using Vik.

### Intervention

To analyze the number of conversations between patients and Vik, we used the data collected since October 2017 to October 2018. We conducted a prospective study by analyzing the users’ data, their usage time, their interest in the various themes proposed, and their level of interactivity.

To analyze the way patients are using Vik, users were asked questions from a survey on a weekly basis since May 2018. These questions concerned various fields (health, food, treatments, and life with the disease; Table 1). The *question of the day* is a subscription from the user who will receive from 1 to 2 times per week an open or a closed question. For open-ended questions, the user had the possibility to read the answers of other community members and to *like* these answers by clicking on *agree* or *disagree*. The sum of these *agree* and *disagree* was defined as the total of a user’s interaction per question of the day.

Finally, we evaluated the general appreciation and level of confidence toward the chatbot with a survey (Table 1).

To evaluate the medication adherence rate of patients using Vik, we implemented a medication reminder function. The user can activate this feature at any time by asking the chatbot, for example, “remind me to take my tamoxifen every day at 4 pm.” The chatbot will then send this person a reminder to take the medication with 3 possible choices for the user: say “yes I took it,” “no I didn’t take it,” or “send me the message in 15 minutes.” We then measured compliance by saving patient responses. The statistical analyses were done using the R software (R Foundation for Statistical Computing *)*. The Student *t* test was used with a 95% CI.

**Table 1:** Examples of questions of the day and questions used for the satisfaction survey.

Thematics	Open-ended questions and closed questions
Diet	What has been the impact of cancer and your treatment on your diet?
Sexuality	Sexuality is often impacted by cancer, what about you? How did you handle it?
Announcement	How did you manage to tell your loved ones, especially your children, about cancer or metastases?
Information	Are you sufficiently informed about the treatments, their benefits, their side effects?
Screening	In your opinion, are breast cancer screening campaigns sufficient?
Clinical trial	Are you currently in a clinical trial?
Satisfaction survey	Do you trust my answers? What is your satisfaction when you chat with me? In your opinion, what could I do to improve myself and be even more useful to you (medical information, functionalities...)? Do you recommend me to your friends? What do I represent or bring to you?

## Results

### Number of Interactions Between Patients and Vik

A total sample of 4737 patients chatted with Vik, (mean age 48 years), 88.90% were female (4211/4737) and 11.10% were male (526/4737; Table 2). Finally, we included 958 patients who answered the various questions.

An average of 132,970 messages exchanged per month was observed between the patients and Vik. We defined the number of messages (a dialogue bubble corresponding to a message) exchanged as the total number of messages received and sent by Vik.

Both patients and relatives used the different features available. They used either direct questions or the answer buttons provided by Vik (Table 3).

We calculated the retention rate for cohorts of patients who started using Vik between February 2018 and October 2018 (n=958). Before that date, we did not have the technological means to measure it. The retention rate is computed for a cohort composed of users who started talking with Vik the same month. It is calculated for each month following the month of arrival of the cohort then. For a given month and a given cohort, the retention rate is the percentage of users in the cohort who were active during the month. A user is considered active from the moment he sent at least one message during over the period. The user retention rate is shown in Table 4. This rate decreases over time, but we observed that some users still chatted with Vik after 8 months.

### The Way Patients Used Vik

The total number of responses to the various questions of the day kept increasing. On average, 60 patients answered those questions: 55.1 open-ended questions and 77.5 closed questions. The total number of interactions averaged 147 per question (for open-ended questions), which makes 2.7 interactions per person per question. User responses for open-ended questions averaged 21.5 words per response or 114.3 characters/response. There were significant differences in the average number of words for each question, according to the themes addressed. We observed a decreasing total number of interactions per question, whereas the average number of words per question increased. The total number of interactions as a function of the average number of words per response does not show a correlation between the 2 factors.

Both total answers and the average number of words per response seemed to increase similarly. Overall, satisfaction with the use of Vik was 93.95% (900/958). Patients regularly left positive comments and recommended Vik to their friends. When we asked them what Vik meant to them and what Vik brought them, 88.00% (843/958) said that Vik provided them with support and helped them follow their treatment effectively (Table 5).

### The Medication Adherence Rate of Patients Using Vik

The number of people using the treatment reminder function was 61. We calculated the average compliance for over 5 weeks (n=33). During week 1, 51% (17/33) of the patients using the feature clicked on the reminder button. We measured this metric over the next 4 weeks.

Table 6 shows that the more the patients chatted with Vik, the more observant they were when they used the feature. The average compliance of patients using the medication reminder feature improved by more than 20% (*P*=.04).

**Table 2:** Demographic data.

Characteristics	Bracket 1	Bracket 2	Bracket 3	Bracket 4	Bracket 5	Bracket 6	Bracket 7
Age groups (years)	13-17	18-24	25-34	35-44	45-54	55-64	65+
Female (n)	55	195	616	768	1427	920	229
Male (n)	0	118	250	95	63	0	0
Proportion (%)	1	7	18	18	32	19	5

**Table 3:** Excerpt from a discussion between a patient and Vik.

Interlocutor	Chat response^a^
Vik	Hello, I'm Vik 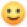 the little robot 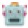 . Are you affected by breast cancer? I'm here to accompany you, answer your questions and more. But before we talk, I wanted to tell you that I respect the European Regulation 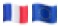 regarding the protection of your data 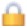 . They are used only to: 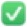 Provide answers adapted to your questions; 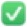 Improve on your use; 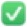 Conduct anonymous statistical studies. 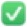 All our conversations are and will remain anonymous. 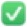 I will never sell your data. You can read here my Terms for more information.
User	I accept
Vik	To chat with me, click on the buttons below and tell me if you are a Patient 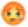 or a Relative 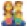 ?
User	Patient 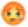
Vik	Alright 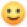 ! Now ask me a question or ask me for example, “what are the questions most frequently asked by other users?”
User	How can you help me Vik?
Vik	Here you can see everything I can do: 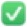 *All topics I can talk about* 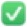 *My features* 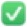 *Manage your subscriptions*
User	Create a reminder
Vik	To create a daily treatment reminder, send me for example: “Remember to take my Nolvadex at 2pm” 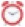 . To create an appointment reminder, send me instead: “Call back my appointment from June 18 at 3pm” 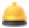
User	How to get psychological help?
Vik	Several people can help you: 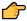 Social worker 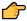 Psycho-oncologists 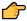 Patient Association
User	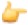 Psycho-oncologists
Vik	The psycho-oncologist 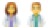 is a psychiatrist or a psychologist specialized in listening and situations of malaise. He is able to identify the distress related to the disease and its treatments. He intervenes on your request or on that of the medical team.

^a^Italicization shows the available options to access other content.

**Table 4:** User retention rate over 8 months (N=956).

Month	Users, n (%)
February	956 (100)
March	691 (72)
April	603 (63)
June	518 (54)
July	427 (45)
August	387 (40)
September	321 (34)
October	296 (31)

**Table 5:** Patient testimonies to the survey: “What does Vik mean to you? What does it bring you?”

Patients	Testimonies
User 1	“Vik brings me information that I didn't know and that the doctor doesn't say.”
User 2	“He supports me. Answers to my questions and not to be alone in my fight. Thanks to vik.”
User 3	“Vik helps me to take my treatments and I really like all the tips and tricks.”
User 4	“Being in a rural area, he allows me to have a contact, like in a group of word, a sharing, and it shows that you are not alone.”
User 5	“Vik represents my reminder every day for my treatment and also offers to great tips.”
User 6	“It allows me to tell my story, to have additional information, to not have not been alone with my questions and just support: when I see Vik it makes me happy, it’s a support.”
User 7	“It’s a virtual help... A quick and succinct source of information...it’s up to us to do research if you need more information. It’s comforting to know that someone can answer us day and night...thank you it's very well done!”
User 8	“It’s like a personal space where I can ask for what I want and have quick answers to the slightest question I have!”

**Table 6:** Observance rate over time (N=33).

Week	Mean observance, n (%)
1	17 (51)
2	20 (61)
3	22 (67)
4	23 (70)
5	25 (76)

## Discussion

### Principal Findings

We aimed to analyze one year of conversations between patients with breast cancer and the chatbot, Vik, and the way they were using it. We observed that some users still chatted with Vik after one year.

Sending a question of the day allowed us to notice that users are more likely to answer multiple choice questions. This is probably due to the fact that it is easier to just click on a button. Answering an open-ended question requires time and reflection. The specific relationship between the response rate to a question and the number of interactions is not apparent. Indeed, there is no *snowball effect* that would show a certain increase in the number of interactions when a question brings many answers.

The questions for which the patients interacted the most (number of likes) and with the most words were the questions dealing with their experiences, such as the announcement to the children or the role of the relatives. The turn of these questions encourages testimony and delivery of their opinion .

On a qualitative level, the question of the day functionality of the chatbot revealed that patients surprisingly shared much with Vik, especially when it came to personal and intimate topics such as sexuality and hair loss. Vik increased patients’ compliance with their treatment thanks to the treatment reminder function. Morawski K et al [18] had already shown that this type of solution improved patient compliance.

Vik is a chatbot, a machine, but patients appreciated discussing with Vik. The results show that it is easy to find support in Vik. Therefore, we believe that a chatbot could allow an effective collection of sensitive, intimate information before the conversation with a doctor in the office. This would probably increase the accuracy of an anamnesis, for example, before a consultation. We can indeed consider that a chatbot can perform this type of method in a way to engage the patients in the treatment of their illness by giving them the opportunity to express themselves about how it impacts their life.

The attention paid to it at that time and the ensuing dialogue can be reassuring and comforting for patients. We think that using a chatbot as an intermediary with physicians facilitates the collection of information. For health care professionals, real-life data feedback is a major asset in the management of their patients. Regular feedback on the progression of the disease and reactions to its treatment provide the physician with a better understanding of patients and their condition. These data can be an aid in the decision-making process.

### General Appreciation

The experience with the chatbot seems very positive to us: patients greatly appreciate the conversational interface and its simplicity. Being able to ask a question and to instantly access a valid answer scientifically and succinctly is a *plus* reported by many patients.

We conducted focus groups with a sample of patients. They reported that the fact that each of Vik’s answer is followed by 2 actions’ contextual information was very popular with users, as it allows them to access the information they would not have thought of. This combination of questions asked and contextual actions (conversation scripting) also pleases, as it makes the interaction more flexible: the user does not systematically have to enter a sentence to access information of interest. We were surprised to find that a real emotional attachment was built up as Vik was used. Some patients confided in other topics than those initially planned when others regularly wished Vik a “good day,” a “good night,” or “thank you.”

### Conclusions

A health care chatbot such as Vik allows patients with breast cancer to have a way to find support and answers to their concerns during their disease. Furthermore, the chatbot Vik improves medication adherence through reminders and educational content, explaining to patients how to take their medication properly, why they have this side effect, and how they can avoid it. New functionalities are planned to confirm Vik as an intermediary between the patient and medical team to provide relevant information to the physicians and enable real-time monitoring.
